# Correction: Białobrzeska, B.; Jasiński, R. Resistance to Abrasive Wear with Regards to Mechanical Properties Using Low-Alloy Cast Steels Examined with the Use of a Dry Sand/Rubber Wheel Tester. *Materials* 2023, *16*, 3052

**DOI:** 10.3390/ma16227152

**Published:** 2023-11-14

**Authors:** Beata Białobrzeska, Robert Jasiński

**Affiliations:** Department of Vehicle Engineering, Faculty of Mechanical Engineering, Wroclaw University of Science and Technology, Wybrzeże Wyspiańskiego 27, 50-370 Wroclaw, Poland; robert.jasinski@pwr.edu.pl

In the original publication [1], there were mistakes in Figure 11b as published including Y scale, value of melt 6 under 200 °C and relationship between melt 1 and melt 2 under all temperature conditions. The corrected Figure 11b appears below. The authors state that the scientific conclusions are unaffected. This correction was approved by the Academic Editor. The original publication has also been updated.

## Figures and Tables

**Figure 11:**
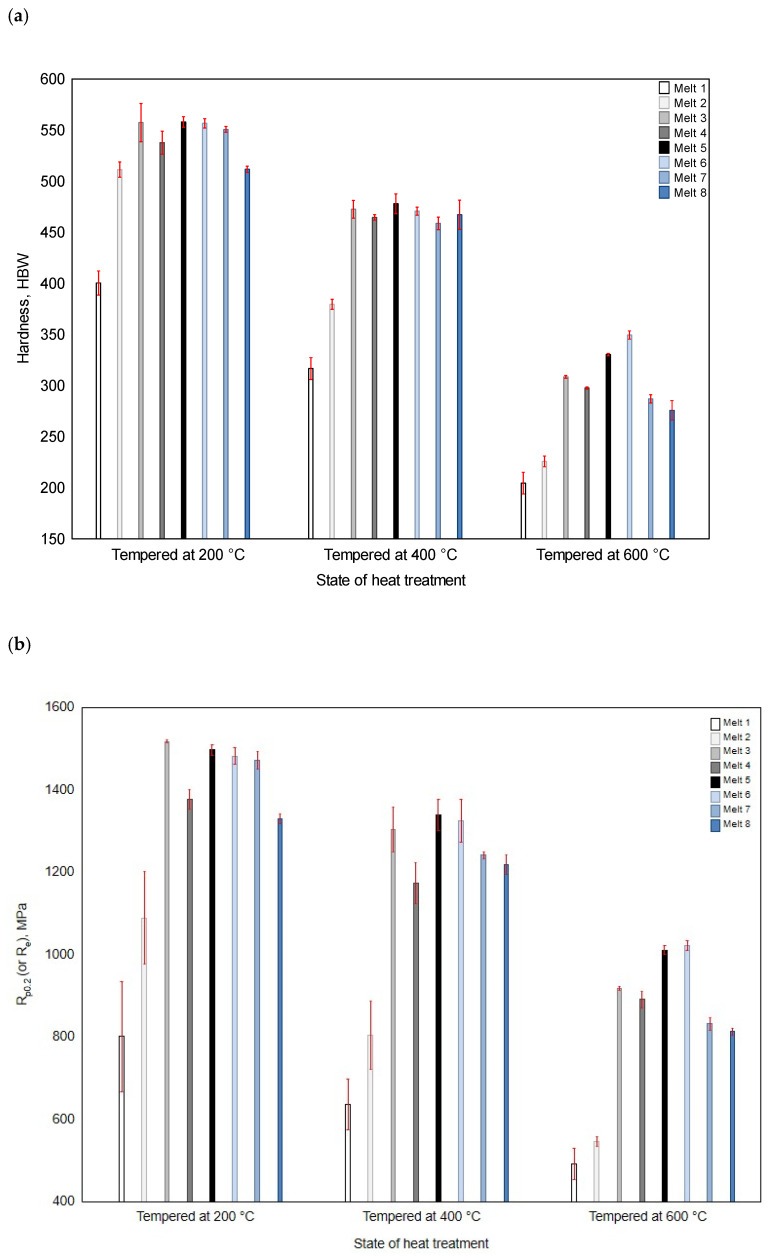
Hardness (**a**) and yield point (**b**) of the analyzed alloys after quenching and tempering at 200, 400 and 600 °C.
